# Word2vec Word Embedding-Based Artificial Intelligence Model in the Triage of Patients with Suspected Diagnosis of Major Ischemic Stroke: A Feasibility Study

**DOI:** 10.3390/ijerph192215295

**Published:** 2022-11-19

**Authors:** Antonio Desai, Aurora Zumbo, Mauro Giordano, Pierandrea Morandini, Maria Elena Laino, Elena Azzolini, Andrea Fabbri, Simona Marcheselli, Alice Giotta Lucifero, Sabino Luzzi, Antonio Voza

**Affiliations:** 1 Emergency Department, IRCCS Humanitas Research Hospital, 20089 Milan, Italy; 2 Department of Biomedical Sciences, Humanitas University, 20072 Milan, Italy; 3 Internal Medicine, Humanitas Research Hospital, 20089 Milan, Italy; 4 Department of Advanced Medical and Surgical Sciences, University of Campania “L. Vanvitelli”, 80138 Naples, Italy; 5 Artificial Intelligence Center, Humanitas Clinical and Research Center—IRCCS, 20089 Milan, Italy; 6 Department of Radiology, IRCCS Humanitas Research Hospital, 20089 Milan, Italy; 7 Health Directorate, IRCCS Humanitas Research Hospital, 20089 Milan, Italy; 8 Department of Systems Medicine, University of Rome “Tor Vergata”, 00133 Rome, Italy; 9 Stroke Unit, IRCCS Humanitas Research Hospital, 20089 Milan, Italy; 10 Department of Clinical-Surgical, Diagnostic and Pediatric Sciences, University of Pavia, 27100 Pavia, Italy; 11 Neurosurgery Unit, Department of Surgical Sciences, Fondazione IRCCS Policlinico San Matteo, 27100 Pavia, Italy

**Keywords:** artificial intelligence, emergency department, major ischemic stroke, word2vec

## Abstract

Background: The possible benefits of using semantic language models in the early diagnosis of major ischemic stroke (MIS) based on artificial intelligence (AI) are still underestimated. The present study strives to assay the feasibility of the word2vec word embedding-based model in decreasing the risk of false negatives during the triage of patients with suspected MIS in the emergency department (ED). Methods: The main ICD-9 codes related to MIS were used for the 7-year retrospective data collection of patients managed at the ED with a suspected diagnosis of stroke. The data underwent “tokenization” and “lemmatization”. The word2vec word-embedding algorithm was used for text data vectorization. Results: Out of 648 MIS, the word2vec algorithm successfully identified 83.9% of them, with an area under the curve of 93.1%. Conclusions: Natural language processing (NLP)-based models in triage have the potential to improve the early detection of MIS and to actively support the clinical staff.

## 1. Introduction

Major ischemic stroke (MIS) affects over 600,000 patients/year, being among the top five causes of death and the first cause of disability in the United States [1]. The MIS evolution time is 10 h on average (range 6–18 h) and it has been estimated that the patient loses 1.9 million neurons for each minute that MIS is untreated [2]. The misdiagnosis of MIS has been associated with false positives (stroke mimics) and false negatives (stroke chameleons) in up to 26% and 43% of cases, respectively [3]. Randomized trials demonstrated that the best outcome is achievable within 4.5 h from the onset of stroke [4,5,6,7,8]. Accordingly, an early and accurate diagnosis of possible MIS patients and their aggressive treatment are mandatory [2,3,9,10,11,12]. While vital, the involvement of human resources such as nurses, neurologists, and radiologists has been reported to act as a time-limiting step in the stroke triage and imaging pathway, especially because this expertise may not be available at all sites or times [2]. These are the main reasons for the increasing interest toward the automatization of the acute management of MIS. Machine learning-based technology has already been used in acute ischemic and hemorrhagic stroke imaging [2,13,14]. However, the semantic models of representation languages and their potential advantages in the optimization of the MIS management still remain largely underestimated.

The aim of the present study is to test the feasibility of the implementation of the word2vec word embedding-based AI model in decreasing the risk of false negatives during the triage of patients with a suspected diagnosis of MIS in the emergency department (ED).

## 2. Methods

The python code for this project is available in the GitHub repository at the following link: https://github.com/pimorandi/MIS_in_ED_admissions (accessed on 14 November 2022).

### 2.1. Data Collection

The study was approved by the Internal Review Board of Humanitas Research Hospital. The patients’ data were retrospectively collected from clinical notes at triage of the ED and referred to the timeframe January 2015–March 2021.

Admission diagnoses were derived from the assigned International Classification of Diseases 9th revision (ICD-9) code after the first visit. The ICD-9 codes specifically selected for their relevance to an MIS were as follows: 434.01 (cerebral thrombosis with cerebral infarction); 434.90 (cerebral artery occlusion, unspecified without mention of cerebral infarction); 434.91 (cerebral artery occlusion, unspecified with cerebral infarction).

### 2.2. Text Preprocessing

The text data underwent “tokenization” consisting of some preprocessing steps to clean and normalize the variables and to separate the paragraphs into words (tokens). Text words were lowercased and normalized through the removal of punctuation, numbers, and non-ASCII characters. A white space character was used as a delimiter for each token, transforming the paragraphs into lists of tokens. Stop words, such as prepositions and articles, were removed to further clean the texts from undesired tokens. The last preprocessing step was the “lemmatization”, aimed at reducing the number of different tokens. The TreeTagger library was used for this step [15].

### 2.3. Text Data Vectorization

**Figure 1:**
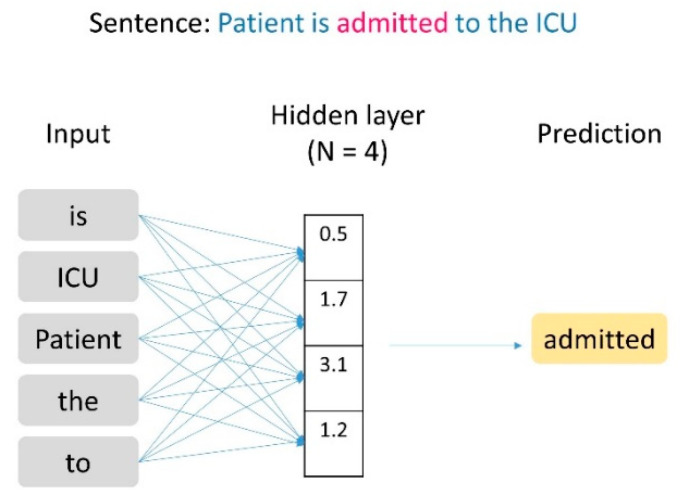
Word2vec embedding model.

The word2vec word-embedding artificial intelligence algorithm was used for the text data vectorization. To produce the embedding, word2vec builds a shallow neural network able to predict a word given its context. The values assumed by the intermediate layer during this prediction are then used as embedding for the given word. The embedding dimension N chosen in this setup is 300, meaning that each word is transposed to a numerical vector of 300 dimensions (Figure 1). The training of the word2vec model was performed using the Gensim Python library [16].

The final vector for each paragraph was obtained averaging the values of the embedding tokens.

### 2.4. Classification and Model Training

Prior to the training, we employed Propensity Score Matching (PSM) [17] to our available confounders (age and gender) to mitigate the bias effect that may skew the results from our model. We devised this latter methodology to retain 100 controls with matched confounders for each MIS sample. The model performances were evaluated via stratified five-fold cross-validation using the scikit-learn Python library [18]. The chosen model was a Gradient Boosted Classification Tree (LightGBM library [19]) and the optimal choice of hyper-parameters was performed using a Bayesian optimization framework (scikit-optimize library) [20]. A logistic regression and a single hidden-layer neural network were also tested, and their performance can be found in Appendix A. The chosen optimization metric was the F1 score since it is a metric particularly fit to deal with imbalanced datasets defined as the harmonic mean of precision and recall. To deal with the data imbalance, different weights were associated with the two classes.

**Figure 2:**
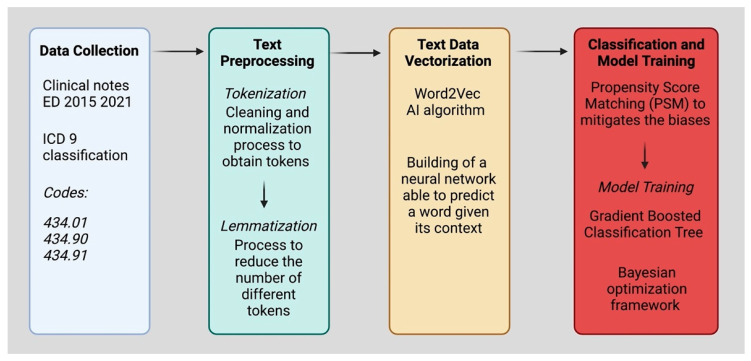
Flowchart of the data collection and processing. ED: emergency department; ICD-9: International Classification of Diseases 9th revision; AI: artificial intelligence.

Figure 2 summarizes the flowchart of the data collection and processing.

## 3. Results

### 3.1. Dataset

The dataset was composed of 305,227 ED admissions divided into 648 MIS and 304,579 non-MIS. The number of female admissions in these two groups is respectively 305 (47.1%) and 148,464 (48.7%). The mean age is 75 (Q1 = 63.9, Q3 = 83.9) for MIS observations and 55 (Q1 = 38.4, Q3 = 73.8) for non-MIS (Table 1).

Since age is strictly correlated with the outcome, the control class had to be subsampled to account for its covariate effect using a PSM technique. The subsampling ratio was 100:1, so for each MIS observation, 100 control observations were selected. After PSM, both gender and age have a non-significant *p*-value related to the outcome. The final cohort is composed of 65,448 observations divided into 648 MIS and 64,800 controls (Table 2).

### 3.2. Classification

**Figure 3:**
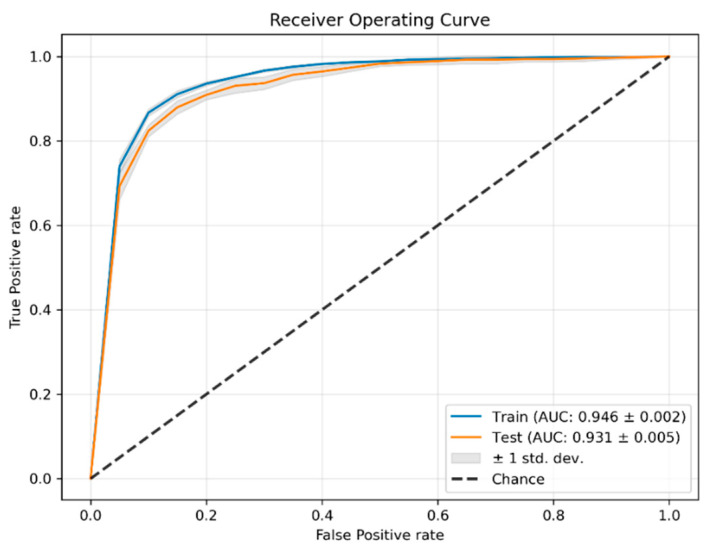
Receiver operating curve in training and testing during cross-validation. The area under the curve (AUC) is 0.946 in training and 0.931 in testing.

In Table 3 is shown the average performance in both the train and test steps of the cross-validation using different metrics. As can be seen, the model is able to learn and generalize to new data. In Figure 3 are plotted the mean ROCs for the train and test steps during cross-validation.

**Figure 4:**
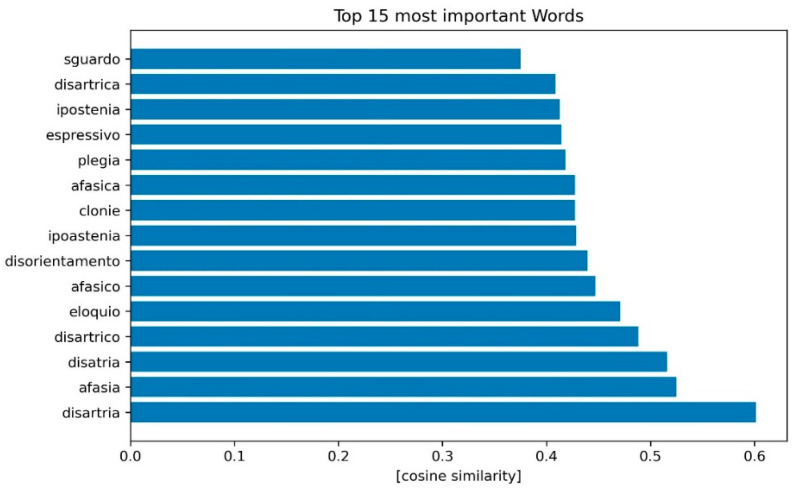
Top words obtained by word embedding.

The word2vec algorithm was able to identify the top 15 words positively correlated to MIS diagnosis using the cosine similarity as a metric between the average stroke patients text vector and the different word vectors. Dysarthria and aphasia were the text words more strongly correlated with the correct diagnosis of MIS (Figure 4).

Afasia or afasico/a: aphasia/aphasic (masculine and feminine adjective); clonie: clonic movements; disartria/disatria: dysarthria, the second word is misspelled/orthographically wrong; disartrico/a: dysarthric (masculine and feminine adjective); disorientamento: disorientation; eloquio: language; espressivo: expressive, a type of aphasic speech (e.g., expressive aphasia); ipostenia/ipoastenia: weakness, the second word is misspelled/orthographically wrong; plegia: plegy; sguardo: gaze.

A brief analysis of the predictive performance of the model stratified per color code (Table 4) shows that for those that are labeled low priority (green) at ED entrance, the model correctly identifies MIS patients when the clinical staff do not; in other words, 61.3% of patients would have been assigned as low priority when in reality they were MIS patients. Of course, due to the low precision for green codes (0.009), the model would trigger far too many false positives to be implemented in an actual clinical setting.

## 4. Discussion

### 4.1. Diagnosis of Major Ischemic Stroke

The present study strived to test the feasibility of the implementation of an NLP-based classification model to optimize the acute management of MIS from triage clinical notes. More than 80% of strokes result from ischemic damage to the brain due to an acute reduction in the blood supply. The goal in the management of acute ischemic stroke is early arterial recanalization to limit the brain damage, since the delay in starting the treatment is associated with worse physical and cognitive outcomes, with a high level of disability and comorbidities [2,21,22]. Although faster triage, improvements in neuroimaging techniques, thrombolysis, and thrombectomy represent the major advances of MIS management, the overall outcome of patients affected by stroke is still largely dependent on a prompt and accurate diagnosis at admission at the ED [12,23,24,25,26,27,28]. Based on our results, keywords-based analysis seems to point to promising results that may yield to a more rapid diagnosis of stroke. The cross-validation performance shows that stroke patients were identified with a recall of 83.9% and an AUC of 93.1%. Dysarthria and aphasia were the text words most importantly correlated with the stroke diagnosis. It is noteworthy that the model was still able to correctly associate a suspected diagnosis of stroke with those misspelled text words that were accidentally recorded during the triage. “Disatria” instead of “disartria”, namely, dysarthric speech, was an example. The practical implication of such a model in daily practice would be non-negligible, since it may contribute to the optimization of the acute management of patients affected by MIS. In a combined vision, where the machine learning models are integrative rather than substitutive of the human resources, the availability of a computer alert generated by the algorithm may be of help to nurses and others to more rapidly recognize those patients suspected to be affected by ischemic stroke. Further algorithms such as those reported in the present study may also be adopted for hemorrhagic stroke, as well as other vascular and non-vascular pathologies of the central nervous system for which a multifactorial genesis is now recognized [29,30,31,32,33].

### 4.2. Word2vec Word Embedding-Based Artificial Intelligence Model

One-hot encoding and word embedding are two of the most popular concepts for vector representation in natural language processing. Word2vec is an algorithm created in 2013 that uses a neural network model to identify words that are associated starting from a big matrix of datasets, and once trained, it can select words with similar meaning from the words surrounding it. It represents each word identified by a list of numbers called vectors. The vectors are selected with a simple mathematical function and share a certain level of semantic similarity between the words associated with those vectors [34]. The choice of word2vec embedding-based algorithm lets us work on a large volume of data in a simple way. This algorithm selects words with intrinsic meaning, starting with a numeric vector obtained from a dependent variable. From the numeric vector (whose length is about 300, established by our team), we process data with a statistic model that can interpret artificial neural networks obtained using the word2vec algorithm. Another algorithm that could be used because of the ease of implementation is “one-hot encoding”, working in a faster way than word embedding: every word has its own value in a vector, but in this process, it loses the semantic meaning of the word in a sentence. One-hot encoding was one of the first techniques used in artificial intelligence models, but with the birth of word embedding, it becomes obsolete, especially in scientific fields. Furthermore, by using a one-hot encoding algorithm, the size of the embedding vector grows with the vocabulary, so it could be difficult to elaborate those data because of the entity of the matrix of embedding obtained, so it does not work well in applications that require a large amount of data. Word2vec, with its implementation, could be a good middle ground because the precision of word embedding depends on the volume of the dataset, so it works well on large datasets obtaining the best word embedding with the smallest matrix. Other algorithms for word embedding include GloVe and FastText. With word2vec, we train a neural network with a single hidden layer to predict a target word based on its context. With FastText, each word is composed of a character n-gram so it can help to generate better word embeddings for rare words or for out-of-vocabulary words; a big limit of this algorithm is that it takes longer to do the embedding and as the dataset grows, the memory required grows too, so in this way is no different to one-hot encoding. The GloVe is a word-embedding technique similar to word2vec, but it differs from it because it is a count-based model instead of a predictive model. In fact, GloVe focuses on word co-occurrences over the whole corpus, while word2vec leverages co-occurrence within a local context (neighboring words). GloVe embeddings relate to the probability that two words appear together. Word-embedding techniques, with respect to count-based methods, are used in different language tasks such as semantic relatedness, synonym detection, concept categorization, and analogy. With word2vec, we observe large improvements in the accuracy at a much lower computational cost, e.g., it takes less than a day to learn high quality. As reported, the need for continuous training of the model, by means of the increase of the data collected from other clinical studies, is a key aspect for the further improvement and optimization of the model itself [35,36].

Lastly, it should be highlighted that the word2vec model has a non-negligible rate of false negatives. Despite this aspect raising concerns about the overall accuracy, it must be stressed that in the authors’ experience, the model was proven to be able to emulate human performance, decreasing the rate of human error, but keeping the clinical biases. For this reason, the model cannot theoretically overcome the overall human performance. We consider this aspect an intrinsic limitation of the model rather than a weakness of the study. Other promising scenarios are worthy of mention since they may prove more accurate in the near future, as suggested by some groups [37,38,39,40].

#### Limitations of the Study

The first limitation of the present study lies in the exclusion of hemorrhagic stroke or TIA, considering only MIS. Furthermore, this word-embedding-based model did not explore the vital signs, which are extremely useful to detect the critical issues of the patient. Using word2vec, we obtained the classification of a word strongly associated with MIS in terms of clinical features, but this algorithm does not work on the definite diagnosis of the disease. With AI models, it would be easy to create a warning signal with those “embedded words”, popping up on computers of triage’s nurses, but the meaning of that “alert” must be evaluated according to the cases. For example, one of the words most associated with stroke diagnosis, according to the word2vec model, is “disorientation”, but only in a few cases is this clinical feature observed in patients. Another limitation of the algorithm is that the detection of true positive cases is not well balanced by the identification of true negative rates. It could overestimate the real impact of the disease in triage. With word2vec, the word embedding obtained using the algorithm is “static”, which means that the model has no awareness of the context in which the word is found. By using recurrent neural networks, the word embedding could become dynamic and more accurate: this new model is able to detect the hidden relationship between inputs as well as to provide a precise sequence prediction of words, giving a high level of accuracy to the results. Future perspectives could involve dynamic models of word embedding such as BERT. Outcome selection is another limitation of this study since we only used the ICD-9 at hospital discharge. Potentially, we would need verified outcomes at 14/28 days and 6 months for every suspected case of MIS at ED admission that was not hospitalized. Those outcomes would further alleviate clinical and other biases.

## 5. Conclusions

The present feasibility study demonstrated that the word2vec word embedding-based AI model was reliable in identifying a suspected diagnosis of MIS during patients’ triage in the ED.

Further studies on larger patient cohorts are mandatory to definitively validate the proposed model.

## Figures and Tables

**Table 1:** Gender and age distribution before PSM.

	Control	MIS	Measure	*p*-Value
Female	148,464 (48.7%)	305 (47.1%)	#	0.13
Age	55 (Q1 = 38.1, Q3 = 73.8)	75 (Q1 = 67.9, Q3 = 83.9)	Years	<<0.001
Admissions	304,579	648	#	

Q1: first quartile, Q3: third quartile, #: not available.

**Table 2:** Gender and age distribution after PSM.

	Control	MIS	Measure	*p*-Value
Female	30,163 (46.5%)	305 (47.1%)	#	0.13
Age	75 (Q1 = 68.3, Q3 = 83.9)	75 (Q1 = 67.9, Q3 = 83.9)	Years	0.86
Admissions	64,800	648	#	

#: not available.

**Table 3:** Performance metrics in the training and testing datasets of the five-fold cross-validation shown as mean (± standard deviation).

	Train		Test	
	Control	MIS	Control	MIS
F1	0.941 (±0.001)	0.137 (±0.002)	0.941 (±0.002)	0.132 (±0.005)
Precision	0.998 (±0.001)	0.074 (±0.001)	0.998 (±0.001)	0.072 (±0.003)
Recall	0.891 (±0.002)	0.878 (±0.005)	0.891 (±0.005)	0.839 (±0.021)
Support	243,663	519	60,916	129

**Table 4:** Cross-validation performances across color codes.

Color Code	Precision		Recall		F1	
	Control	MIS	Control	MIS	Control	MIS
Red	0.987	0.138	0.721	0.834	0.833	0.237
Yellow	0.995	0.110	0.827	0.864	0.903	0.195
Green	0.999	0.009	0.927	0.613	0.961	0.018

## Data Availability

Not applicable.

## Appendix A

In addition to the Gradient Boosted Trees, the model selection process also considered a logistic regression and a feed-forward neural network. The logistic regression underwent the same hyper-parameter optimization described in Section 2.4. The neural network is composed of a single hidden layer whose dimensionality has been set by manual investigation to six neurons. The performances are shown below.

**Table A1:** Neural network performances.

	Train		Test	
	Control	MIS	Control	MIS
F1	0.959 (±0.005)	0.187 (±0.018)	0.957 (±0.007)	0.175 (±0.018)
Precision	0.999 (±0.001)	0.109 (±0.011)	0.998 (±0.001)	0.098(±0.011)
Recall	0.921 (±0.010)	0.959 (±0.011)	0.920 (±0.013)	0.847 (±0.029)
Support	243,663	519	60,916	129

**Table A2:** Logistic regression performances.

	Train		Test	
	Control	MIS	Control	MIS
F1	0.955 (±0.004)	0.177 (±0.011)	0.954 (±0.004)	0.169 (±0.011)
Precision	0.999 (±0.001)	0.098 (±0.007)	0.998 (±0.001)	0.094(±0.007)
Recall	0.914 (±0.008)	0.923 (±0.006)	0.914 (±0.009)	0.879 (±0.019)
Support	243,663	519	60,916	129

As can be seen, both of these models seem to lead to better classifications compared to the Gradient Boosted Trees, but a more in-depth analysis of the performances across color codes shows that the ensemble method generalizes better to low priority code (green).

**Table A3:** Neural network performance across color codes.

Color Code	Precision		Recall		F1	
	Control	MIS	Control	MIS	Control	MIS
Red	0.990	0.163	0.762	0.869	0.861	0.275
Yellow	0.996	0.134	0.861	0.871	0.923	0.233
Green	0.999	0.013	0.953	0.568	0.976	0.026

**Table A4:** Logistic regression performance across color codes.

Color Code	Precision		Recall		F1	
	Control	MIS	Control	MIS	Control	MIS
Red	0.995	0.163	0.743	0.939	0.851	0.279
Yellow	0.997	0.126	0.846	0.897	0.915	0.221
Green	0.999	0.013	0.952	0.568	0.975	0.026

## References

[1] Go A.S., Mozaffarian D., Roger V.L., Benjamin E.J., Berry J.D., Blaha M.J., Dai S., Ford E.S., Fox C.S., Franco S. (2014). Heart disease and stroke statistics--2014 update: A report from the American Heart Association. Circulation.

[2] Saver J.L. (2006). Time is brain—Quantified. Stroke.

[3] Liberman A.L., Prabhakaran S. (2017). Stroke Chameleons and Stroke Mimics in the Emergency Department. Curr. Neurol. Neurosci. Rep..

[4] Levine D.A., Wadley V.G., Langa K.M., Unverzagt F.W., Kabeto M.U., Giordani B., Howard G., Howard V.J., Cushman M., Judd S.E. (2018). Risk Factors for Poststroke Cognitive Decline: The REGARDS Study (Reasons for Geographic and Racial Differences in Stroke). Stroke.

[5] Saver J.L., Fonarow G.C., Smith E.E., Reeves M.J., Grau-Sepulveda M.V., Pan W., Olson D.M., Hernandez A.F., Peterson E.D., Schwamm L.H. (2013). Time to Treatment With Intravenous Tissue Plasminogen Activator and Outcome From Acute Ischemic Stroke. JAMA.

[6] Emberson J., Lees K.R., Lyden P., Blackwell L., Albers G., Bluhmki E., Brott T., Cohen G., Davis S., Donnan G. (2014). Effect of treatment delay, age, and stroke severity on the effects of intravenous thrombolysis with alteplase for acute ischaemic stroke: A meta-analysis of individual patient data from randomised trials. Lancet.

[7] Tawil S.E., Muir K.W. (2017). Thrombolysis and thrombectomy for acute ischaemic stroke. Clin. Med..

[8] Albers G.W., Marks M.P., Kemp S., Christensen S., Tsai J.P., Ortega-Gutierrez S., McTaggart R.A., Torbey M.T., Kim-Tenser M., Leslie-Mazwi T. (2018). Thrombectomy for Stroke at 6 to 16 Hours with Selection by Perfusion Imaging. N. Engl. J. Med..

[9] Fugate J.E., Rabinstein A.A. (2015). Absolute and Relative Contraindications to IV rt-PA for Acute Ischemic Stroke. Neurohospitalist.

[10] Ekundayo O.J., Saver J.L., Fonarow G.C., Schwamm L.H., Xian Y., Zhao X., Hernandez A.F., Peterson E.D., Cheng E.M. (2013). Patterns of emergency medical services use and its association with timely stroke treatment: Findings from Get With the Guidelines-Stroke. Circ. Cardiovasc. Qual. Outcomes.

[11] Jia J., Band R., Abboud M.E., Pajerowski W., Guo M., David G., Mechem C.C., Messé S.R., Carr B.G., Mullen M.T. (2017). Accuracy of Emergency Medical Services Dispatcher and Crew Diagnosis of Stroke in Clinical Practice. Front. Neurol..

[12] Schwamm L.H., Wu O., Song S.S., Latour L.L., Ford A.L., Hsia A.W., Muzikansky A., Betensky R.A., Yoo A.J., Lev M.H. (2018). Intravenous thrombolysis in unwitnessed stroke onset: MR WITNESS trial results. Ann. Neurol..

[13] Murray N.M., Unberath M., Hager G.D., Hui F.K. (2020). Artificial intelligence to diagnose ischemic stroke and identify large vessel occlusions: A systematic review. J. Neurointerv. Surg..

[14] Soun J.E., Chow D.S., Nagamine M., Takhtawala R.S., Filippi C.G., Yu W., Chang P.D. (2021). Artificial Intelligence and Acute Stroke Imaging. AJNR Am. J. Neuroradiol..

[15] Schmidt H. (2013). Probabilistic part-of-speech tagging using decision trees. New Methods in Language Processing.

[16] Rehurek R., Sojka P. (2011). Gensim–python framework for vector space modelling. NLP Cent. Fac. Inform. Masaryk. Univ. Brno Czech Repub..

[17] Kline A., Luo Y. (2022). PsmPy: A Package for Retrospective Cohort Matching in Python. Annu. Int. Conf. IEEE Eng. Med. Biol. Soc..

[18] Pedregosa F., Varoquaux G., Gramfort A., Michel V., Thirion B., Grisel O., Blondel M., Louppe G., Prettenhofer P., Weiss R. (2011). Scikit-learn: Machine Learning in Python. J. Mach. Learn. Res..

[19] Ke G., Meng Q., Finley T., Wang T., Chen W., Ma W., Ye Q., Liu T.-Y. LightGBM: A Highly Efficient Gradient Boosting Decision Tree. Proceedings of the 31st International Conference on Neural Information Processing Systems (NIPS).

[20] Tim Head, MechCoder, Gilles Louppe, Iaroslav Shcherbatyi, fcharras, Zé Vinícius, cmmalone, Christopher Schröder, nel215, Nuno Campos Scikit-optimize/scikit-optimize: v0.5.2. https://zenodo.org/record/1207017#.Y3eIH3bMJPY..

[21] Prabhakaran S., Ruff I., Bernstein R.A. (2015). Acute stroke intervention: A systematic review. JAMA.

[22] Arenillas J.F., Cortijo E., García-Bermejo P., Levy E.I., Jahan R., Liebeskind D., Goyal M., Saver J.L., Albers G.W. (2018). Relative cerebral blood volume is associated with collateral status and infarct growth in stroke patients in SWIFT PRIME. J. Cereb. Blood Flow Metab..

[23] Oostema J.A., Chassee T., Baer W., Edberg A., Reeves M.J. (2019). Brief Educational Intervention Improves Emergency Medical Services Stroke Recognition. Stroke.

[24] Gorchs-Molist M., Solà-Muñoz S., Enjo-Perez I., Querol-Gil M., Carrera-Giraldo D., Nicolàs-Arfelis J.M., Jiménez-Fàbrega F.X., Pérez de la Ossa N. (2020). An Online Training Intervention on Prehospital Stroke Codes in Catalonia to Improve the Knowledge, Pre-Notification Compliance and Time Performance of Emergency Medical Services Professionals. Int. J. Environ. Res. Public Health.

[25] Oostema J.A., Nasiri M., Chassee T., Reeves M.J. (2014). The quality of prehospital ischemic stroke care: Compliance with guidelines and impact on in-hospital stroke response. J. Stroke Cerebrovasc. Dis..

[26] Lin C.B., Peterson E.D., Smith E.E., Saver J.L., Liang L., Xian Y., Olson D.M., Shah B.R., Hernandez A.F., Schwamm L.H. (2012). Emergency medical service hospital prenotification is associated with improved evaluation and treatment of acute ischemic stroke. Circ. Cardiovasc. Qual. Outcomes.

[27] Yperzeele L., Van Hooff R.J., De Smedt A., Valenzuela Espinoza A., Van de Casseye R., Hubloue I., De Keyser J., Brouns R. (2014). Prehospital stroke care: Limitations of current interventions and focus on new developments. Cerebrovasc. Dis..

[28] Brice J.H., Griswell J.K., Delbridge T.R., Key C.B. (2002). Stroke: From recognition by the public to management by emergency medical services. Prehosp. Emerg. Care.

[29] Bellantoni G., Guerrini F., Del Maestro M., Galzio R., Luzzi S. (2019). Simple schwannomatosis or an incomplete Coffin-Siris? Report of a particular case. eNeurologicalSci.

[30] Luzzi S., Del Maestro M., Elbabaa S.K., Galzio R. (2020). Letter to the Editor Regarding “One and Done: Multimodal Treatment of Pediatric Cerebral Arteriovenous Malformations in a Single Anesthesia Event”. World Neurosurg..

[31] Luzzi S., Del Maestro M., Galzio R. (2019). Letter to the Editor. Preoperative embolization of brain arteriovenous malformations. J. Neurosurg..

[32] Campanella R., Guarnaccia L., Cordiglieri C., Trombetta E., Caroli M., Carrabba G., La Verde N., Rampini P., Gaudino C., Costa A. (2020). Tumor-Educated Platelets and Angiogenesis in Glioblastoma: Another Brick in the Wall for Novel Prognostic and Targetable Biomarkers, Changing the Vision from a Localized Tumor to a Systemic Pathology. Cells.

[33] Luzzi S., Crovace A.M., Lacitignola L., Valentini V., Francioso E., Rossi G., Invernici G., Galzio R.J., Crovace A. (2018). Engraftment, neuroglial transdifferentiation and behavioral recovery after complete spinal cord transection in rats. Surg. Neurol. Int..

[34] Mikolov T., Chen K., Corrado G.s., Dean J. Efficient Estimation of Word Representations in Vector Space. Proceedings of the Workshop at ICLR.

[35] Powers W.J., Rabinstein A.A., Ackerson T., Adeoye O.M., Bambakidis N.C., Becker K., Biller J., Brown M., Demaerschalk B.M., Hoh B. (2018). 2018 Guidelines for the Early Management of Patients With Acute Ischemic Stroke: A Guideline for Healthcare Professionals From the American Heart Association/American Stroke Association. Stroke.

[36] Jiang F., Jiang Y., Zhi H., Dong Y., Li H., Ma S., Wang Y., Dong Q., Shen H., Wang Y. (2017). Artificial intelligence in healthcare: Past, present and future. Stroke Vasc. Neurol..

[37] Wang J., Zhao C., He S., Gu Y., Alfarraj O., Abugabah A. (2022). LogUAD: Log Unsupervised Anomaly Detection Based on Word2Vec. Comput. Syst. Sci. Eng..

[38] Pu B., Li K., Li S., Zhu N. (2021). Automatic Fetal Ultrasound Standard Plane Recognition Based on Deep Learning and IIoT. IEEE Trans. Ind. Inform..

[39] Wang J., Yang Y., Wang T., Sherratt R.S., Zhang J. (2020). Big Data Service Architecture: A Survey. J. Internet Technol..

[40] Duan M., Li K., Liao X., Li K. (2018). A Parallel Multiclassification Algorithm for Big Data Using an Extreme Learning Machine. IEEE Trans. Neural Netw. Learn. Syst..

